# Evaluating the efficiency of enzyme accelerated CO_2_ capture: chemical kinetics modelling for interpreting measurement results

**DOI:** 10.1080/14756366.2020.1864631

**Published:** 2021-01-12

**Authors:** Lorenzo Parri, Ada Fort, Anna Lo Grasso, Marco Mugnaini, Valerio Vignoli, Clemente Capasso, Sonia Del Prete, Maria Novella Romanelli, Claudiu T. Supuran

**Affiliations:** a Department of Information Engineering and Mathematics, University of Siena, Siena, Italy; b Department of Biology, Agriculture and Food Sciences, CNR -Institute of Biosciences and Bioresources (IBBR-CNR), Napoli, Italy; c Department of Neurosciences, Psychology, Drug Research and Child Health (NEUROFARBA), University of Florence, Sesto Fiorentino, Italy

**Keywords:** Gas measurement system, chemical system measurement, carbonic anhydrase, kinetics modelling

## Abstract

In this paper, the efficiency of the carbonic anhydrase (CA) enzyme in accelerating the hydration of CO_2_ is evaluated using a measurement system which consists of a vessel in which a gaseous flow of mixtures of nitrogen and CO_2_ is bubbled into water or water solutions containing a known quantity of CA enzyme. The pH value of the solution and the CO_2_ concentration at the measurement system gas exhaust are continuously monitored. The measured CO_2_ level allows for assessing the quantity of CO_2_, which, subtracted from the gaseous phase, is dissolved into the liquid phase and/or hydrated to bicarbonate. The measurement procedure consists of inducing a transient and observing and modelling the different kinetics involved in the steady-state recovery with and without CA. The main contribution of this work is exploiting dynamical system theory and chemical kinetics modelling for interpreting measurement results for characterising the activity of CA enzymes. The data for model fitting are obtained from a standard bioreactor, in principle equal to standard two-phase bioreactors described in the literature, in which two different techniques can be used to move the process itself away from the steady-state, inducing transients.

## 1. Introduction

The characterisation of complex dynamical phenomena requires sophisticated and advanced measurement procedures and systems. This is especially true for chemical/biochemical processes (see, e.g.^1–6^) where many different influence quantities may play a role in the measurement outcomes. In this context, it is of the utmost importance modelling the dynamics to plan, design, or optimise measurements. An efficient modelling allows us to understand the influence quantities, retrieve the meaningful parameters, overcome some limitations of the measurement system, and unravel the most important features of the phenomenon of interest.

In this paper, the problem of proving and measuring the efficiency of carbonic anhydrase (CA) enzymes in accelerating the rate of CO_2_ hydration is dealt with an *ad hoc* measurement system, based on the reaction kinetics real-time monitoring and the use of a dynamic model of the involved chemical reactions^7^^,^^8^.

CAs catalyse the physiologically crucial reversible reaction of the carbon dioxide (CO_2_) hydration to bicarbonate (HCO_3_^−^) and protons (H^+^) according to the following chemical reaction^9^^,^^10^: CO_2_ + H_2_O ⇋ HCO_3_^−^ + H^+^. In the last years, CA has been considered one of the most promising biocatalysts for CO_2_ sequestration technology to counteract the accumulation of CO_2_ gas in the atmosphere^11^, which is the leading cause of global climate changes. CA enzyme-based techniques exploit highly efficient biological CO_2_ mechanisms, which are naturally occurring in living organisms and are involved in many physiological CO_2_ reactions such as respiration in mammalian cells or photosynthesis in plant cells. The adoption of a CO_2_-catalyzing enzyme allows the development of an efficient and eco-friendly ‘bio-mimic’ CO_2_ capture system of potential interest in various applications in the framework of environmental control. Recently, Capasso et al. focussed their scientific attention on the presence of α-CAs in thermophilic microorganisms, such as *Sulfurihydrogenibium yellowstonense* and *Sulfurihydrogenibium azorense*^12^^,^^13^. The study of the biochemical and physical properties of the ‘extreme’ bacterial CAs has led to the discovery of molecular features, which make them different from those of the mesophilic counterpart allowing their use in biotechnological fields generally characterised by conditions deleterious for the enzyme activity^12^^,^^13^. Moreover, the X-ray tridimensional structures of the two ‘extremo-CAs’ indicated with the acronyms SspCA and SazCA provided the rationale at molecular level for their thermostability.

Recently, it has been realised a three-phase bioreactor (gas, liquid, and solid), which was filled with the recombinant SspCA immobilised on polyurethane (PU)^14^. The results obtained using the lab-scale bioreactor showed that the immobilised PU-SspCA is capable of converting CO_2_ from a gas mixture, whose initial concentration was 20%. Russo et al., using the SspCA covalently immobilised on paramagnetic Fe_3_O_4_ nanoparticles via carbodiimide activation of the enzyme and the protocol based on CO_2_ absorption experiment in a stirred cell apparatus, determined the kinetics of the immobilised SspCA for the CO_2_ hydration reaction^15^. Abdelrahim et al.^16^ provided an innovative concept for the removal of CO_2_ from flue gas streams, using biomimetic SILMs (Supported Ionic Liquid Membranes) containing SspCA that enhances the selective transport of CO_2_.

As described in the literature, there are some disadvantages using the free enzyme in solution because the repeatable usage of the biocatalyst is limited, and generally it is not possible to recover the catalyst from the reaction mixture. Fortunately, these disadvantages can be eliminated immobilising the enzyme on specific supports^17^ even if it may discourage the extensive utilisation of enzymes in industrial applications because of the high costs connected to the biocatalyst production and purification, and the expenses for the preparation of the immobilisation support. This limitation can be easily overcome by the in vivo immobilisation, which consists in the overexpression of protein directly onto the surface of bacterial hosts^11^.

In this paper, which follows the work presented in^18^, both the thermostable CA (SspCA) immobilised on the external surface of *Escherichia coli* cells^11^, and the commercial CA from bovine erythrocytes (Sigma-Aldrich C-3934), were used to explore the enzymatic CO_2_ conversion into bicarbonate. The CAs efficiency in accelerating the hydration of CO_2_ is assessed using a measurement system, consisting of a vessel in which a constant gaseous flow of a mixture of air and CO_2_ is bubbled into a water solution containing a known quantity of the SspCA. The pH value is continuously controlled by a pH probe, whereas an IR based sensor controls the CO_2_ concentration at the gas exhaust of the system. The measured CO_2_ concentration allows for assessing the quantity of CO_2_, which is subtracted from the gaseous phase, dissolved into the liquid phase and/or hydrated. The measurement procedure consists of inducing a transient, exploiting a buffer solution or changing the input CO_2_ concentration, and observing the different kinetics involved in the recovery of the steady-state with and without CA.

## 2. Materials and methods

### 2.1. Measurement system

**Figure 1.**
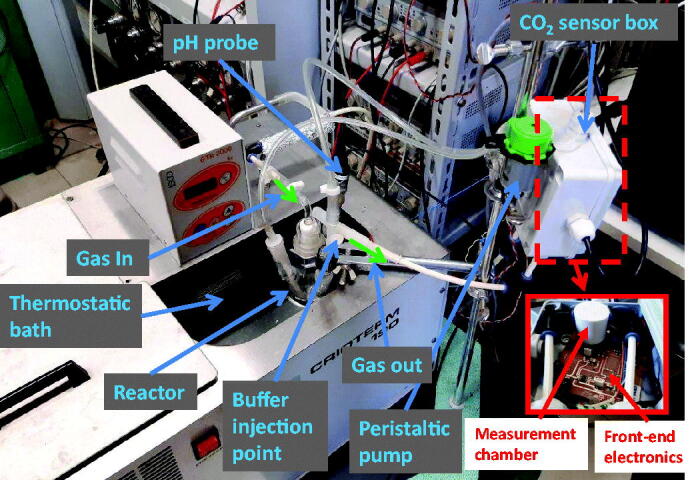
Measurement system setup.

**Figure 2.**
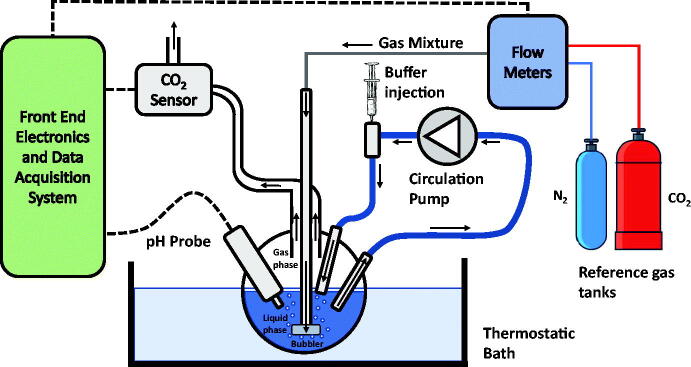
Reactor.

The measurement system, whose complete setup is shown in Figure 1, is based on the reactor shown in Figure 2. In the vessel, which is hosted in a thermostatic bath containing 100 ml of pure water or pure water and a known quantity of carbonic anhydrase enzyme, a constant and controlled flow (200 ml/min) of a mixture of synthetic air and CO_2_ is continuously injected. The flow is set by a digitally controlled gas flow meter (BronkHorst F-201C). A peristaltic pump is used to circulate the fluid phase with a flow of 100 ml/min. The vessel exhaust embeds a non-dispersive infra-red (NDIR) CO_2_ sensor, the IRCA1 from Alphasense, with a declared accuracy of about 2500 ppm. This sensor^19^ is composed of a lamp and two infra-red pyroelectric detectors. The light emitted from the lamp has a spectrum that includes the CO_2_ absorption peak in the infra-red domain. The lamp must be externally driven by a signal that periodically turns on and off the light to avoid sensor overheating. The same light passes through the gas sample and a reference channel. The two detector signals, one per channel, are acquired and, taking the difference between the two signals, the absorbance due to CO_2_ is calculated. The CO_2_ concentration can be obtained from the absorbance according to the Beer-Lambert law.

An electrochemical pH probe (Jumo 201005) is immersed in the water solution. The output voltage of the probe is read using a 6½ digital multimeter (Keysight Technologies AG34410A), whereas the NDIR sensor output is acquired with an *ad hoc* designed front end electronics and a National Instruments 16 bits DAQ board (NI PCI6259). The gas sensor NDIR lamp is operated with a 2 Hz square-wave signal obtained from an arbitrary waveform generator (Keysight Technologies AWG33220A). The whole system is managed by a PC using a LabView Virtual Instrument. Both the pH and the CO_2_ signals are acquired with a sampling time of 0.5 s. An additional aperture of the vessel allows for injecting a buffer solution (tris-hydroxymethyl-aminomethane, 10 mM, pH 8.3 @ *T* = 25 °C).

### 2.2. Chemical system dynamical model

The relevant reactions occurring in the reactor are reported in the following^20–25^:

(1) $$CO_{2gas}\overset{k_{f0}}{\to }CO_{2}$$

(2) $$H_{2}O+CO_{2}\begin{array}{c} \overset{k_{f1}}{\to }\\ \underset{k_{b1}}{\leftarrow } \end{array}H_{2}CO_{3}$$

(3) $$H_{2}CO_{3}\begin{array}{c} \overset{k_{f2}}{\to }\\ \underset{k_{b2}}{\leftarrow } \end{array}HCO_{3}^{-}+\;H^{+}$$

where *k_fi_* and *k_bi _*[1/s] are the rate constants of the direct and reverse reactions, *i* = 0–2.

The first reaction is the solution of gaseous CO_2_ in water, which forms aqueous CO_2_ (1). The second is the reaction of aqueous CO_2_ with water to form carbonic acid (2). In the next step (reaction 3), carbonic acid dissociates to ions.

In the general case, the kinetics of these three reactions can be used to derive the dynamical model of the solution of carbon dioxide in water, and to simulate the behaviour of the dissolved CO_2_ concentration, [CO_2_].

(4) $$\frac{d[CO_{2}]}{dt}\;=\;k_{f0}({\left[CO_{2}\right]}^{*}-\;[CO_{2}])\text{ − }k_{f1}[CO_{2}]+k_{b1}[H_{2}CO_{3}]$$

(5) $$\frac{d[H_{2}CO_{3}]}{dt}=k_{f1}[CO_{2}]-{(k}_{b1}+k_{f2})[H_{2}CO_{3}]+k_{b2}[HCO_{3}^{-}]\;$$

(6) $$\frac{d[{HCO_{3}}^{-}]}{dt}=\frac{d[H^{+}]}{dt}=k_{f2}[H_{2}CO_{3}]-k_{b2}[HCO_{3}^{-}]$$

In all these equations, [*X*] is the concentration of the species X. $\frac{d\left[CO_{2}\right]}{dt}$ is the rate of aqueous CO_2_ dissolving in water from the gaseous phase, and [*CO_2_*]* is the saturation value of the overall concentration of aqueous CO_2_ concentration, due to Henry’s law. [*CO_2_*]* depends on temperature, pressure and on the concentration of gaseous CO_2_. In (2) and (3) a first order kinetics was assumed on the basis of the experimental observations, which are supported by the satisfactory behaviour of the developed model.

The model described by Equations (4)–(6) can be simplified according to the following considerations and assumptions. The reaction rate constants *k_f1_* and *k_b1_* magnitude order is 10^−2 ^s^−1^ and 10^0^ s^−1^, respectively, whereas *k_f2_* and *k_b2_* results in general much larger^20^^,^^21^. Therefore, the first two reactions, Equations (4) and (5), are the rate limiting steps, and the kinetics of these two reactions can be used to derive the dynamical model of the solution of carbon dioxide in water, and to simulate the behaviour of the dissolved CO_2_ concentration, [*CO_2_*]. This is the assumption commonly made in literature^20^.

In detail, since reaction (3) is very fast with respect to reactions (1) and (2), it is assumed to reach the equilibrium instantaneously, so that:

(7) $$[HCO_{3}^{-}]=K_{eq}[H_{2}CO_{3}],$$

where $K_{eq}=\frac{k_{f2}}{k_{b2}}$ is the equilibrium constant of Equation (3). Note that the pH value can be evaluated from the concentration $\left[HCO_{3}^{-}\right]$, considering:

(8) $$pH≃-\log_{10}([HCO_{3}^{-}])=-\log_{10}\left(K_{eq}[H_{2}CO_{3}]\right)$$

Therefore, a system of two first order linear equations can be used to describe the concentration of aqueous CO_2_:

(9) $$\begin{array}{c} \frac{d\left[CO_{2}\right]}{dt}\;=\frac{k_{b1}}{K_{eq}}\left[HCO_{3}^{-}\right]-k_{f1}\left[CO_{2}\right]+k_{f0}({\left[CO_{2}\right]}^{*}-\;\left[CO_{2}\right])\\ \frac{d[HCO_{3}^{-}]}{dt}\;=k_{f1}K_{eq}\left[CO_{2}\right]-k_{b1}\left[HCO_{3}^{-}\right] \end{array}$$

In the presence of the carbonic anhydrase enzyme, the mechanism of hydration of CO_2_ changes completely, and reactions (2) and (3) are replaced by a different reaction route^21^:

(10) $$\begin{array}{c} \mathrm{EZn}H_{2}O\;\overset{}{↔}E\mathrm{ZnO}H^{-}+H^{+}\\ \mathrm{EZnO}H^{-}+CO_{2}\overset{}{↔}E\mathrm{ZnHC}O_{3}^{-}\\ \mathrm{EZnHC}O_{3}^{-}+H_{2}O\overset{}{↔}EZnH_{2}O+HCO_{3}^{-} \end{array}$$

where *EZn* indicates the carbonic anhydrase enzyme.

The kinetics of the overall reaction can be modelled by a differential equation system with the same form as in (4)–(6), but with different reaction rate constants.

To relate the measured CO_2_ gas concentration to the dissolved CO_2_ concentration, the following considerations are made. The gas CO_2_ is bubbled into the water solution, a part of the CO_2_ is solved, and the remaining part, by the bubbles, flows out. Defining *φ*_ΙΝ_ as the CO_2_ gas flow (which is a known quantity), bubbled in the water (injected into the system), $\varphi_{solved}$ as the CO_2_ flow that is solved into the liquid phase and which becomes aqueous, and $\varphi_{OUT}$ the fraction of the CO_2_ flow that remains gaseous, we have:

(11) $$\begin{array}{c} \varphi_{\mathrm{OUT}}=\;\varphi_{IN}-\varphi_{\mathrm{solved}}=\\ =\varphi_{IN}-\frac{d{\left[CO_{2}\right]}_{\mathrm{gas}}}{dt}Vol=\\ =\varphi_{IN}-\left\{k_{f0}\left({\left[CO_{2}\right]}^{*}-\;\left[CO_{2}\right]\right)\right\}\;\mathrm{Vol} \end{array}$$

where *Vol* represents the volume of water.

Therefore:

(12) $$[CO_{2}]=\frac{\varphi_{\mathrm{OUT}}-{\;\varphi }_{IN}}{k_{f0}Vol}+{\left[CO_{2}\right]}^{*}$$

Finally, the output flow of CO_2_, $\varphi_{OUT}$, is proportional to the measured concentration [*CO*_2_]_gasOUT_, that can be written as follows:

(13) $${\left[CO_{2}\right]}_{\mathrm{gas}\;\mathrm{OUT}}=\frac{\left({\varphi_{IN}-\varphi }_{\mathrm{solved}}\right)}{\varphi_{IN}}{\left[CO_{2}\right]}_{\mathrm{gas}\;}$$

where ${\left[CO_{2}\right]}_{gas}$ is the concentration of CO_2_ in the injected flow.

In the next section it will be shown with specific tests that even if the complete model is in principle more effective and accurate in describing the kinetics of the CO_2_ exchange between the two phases, in general the simplified model is accurate enough to describe the dynamic behaviour of the system.

### 2.3. Measurement procedure

The measurement procedure is based on the monitoring of ${\left[CO_{2}\right]}_{gasOUT}$ , i.e., the concentration of carbon dioxide in the output flow, and of the pH value of the liquid phase during a transient in the presence of different quantities of CA enzyme. The reason behind this approach is that the transient response behaviour can give information about the rate constants of the reactions described in the previous section, and can be therefore used to characterise the efficiency of CA enzymes.

Two different procedures were used to induce a transient in the system.

The first procedure is the classical method proposed in the literature for this type of measurement, which is based on the use of a buffer solution to suddenly change the value of the pH. In this case the transient is started by injecting a fixed amount of a buffer solution in the liquid phase (distilled water), with and without the enzyme, which was before brought at the steady state bubbling a constant flow of the CO_2_ mixture for a sufficient time. Therefore, before the injection, the pH value and the concentration of aqueous CO_2_ were stable with [*CO_2_*] equal to ${\left[CO_{2}\right]}^{*}$. The injection causes a sudden change in the concentration of [*H^+^*] and, due to the fast reaction (3), a corresponding change in $\left[HCO_{3}^{-}\right]$, which can be considered step changes. The dynamical system evolves starting from this step change according to the models presented in the previous section.

In this work also a second way to induce a transient was adopted, which doesn’t require a buffer solution. At the basis of the developed models there is the assumption that the buffer does not play a role in reactions (1)–(3): the use of this alternative approach makes the assumption no more necessary. In other words, with this technique we are sure that the reactions (1)–(3), (10) are sufficient to describe what happens in the bioreactor. In detail, the transient is induced by step-changing the CO_2_ concentration in the gas mixture bubbled in the liquid phase, inducing as a consequence an abrupt change in the pH value. In detail, before starting the measurement, in the reactor a constant flow of N_2_ is bubbled for a time long enough to obtain a stable pH measurement. After that, the gas mixture composition is changed, while keeping constant the total flow, introducing a known concentration of CO_2_. This change triggers a transient both in the CO_2_ concentration at the output of the reactor and in pH value of the liquid phase. The above operations can be repeated alternating phases of pure N_2_ with phases in which mixtures of N_2_ and different concentrations of CO_2_ are bubbled in the reactor. These operations are performed continuously, using the same liquid sample in the reactor, with the advantage of increasing the time efficiency of the measurement procedure.

**Figure 3.**
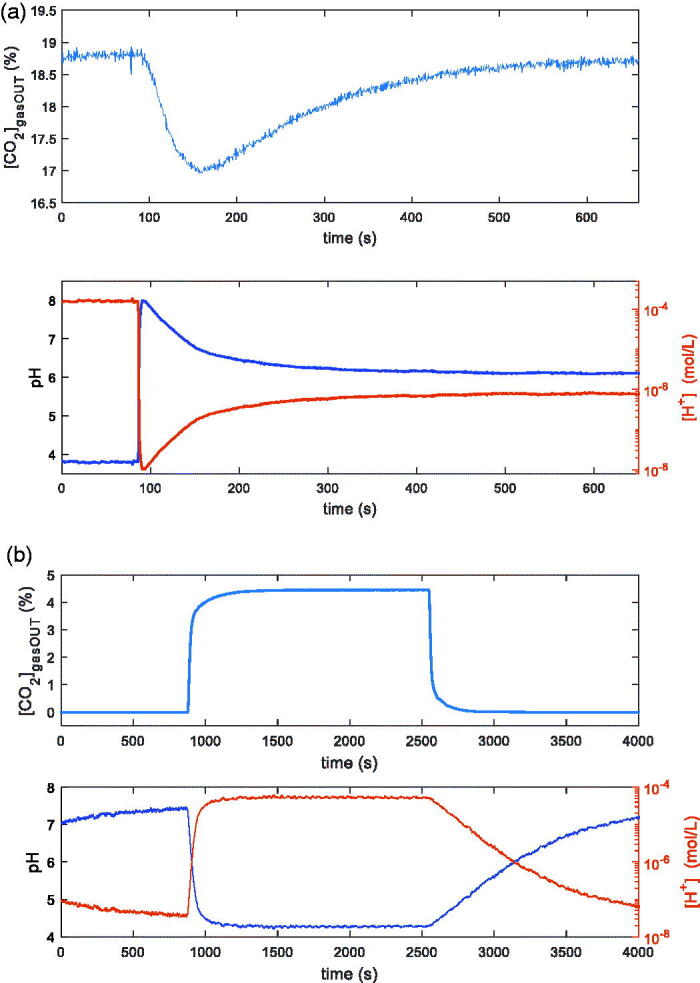
Examples of transients induced in two different ways in the reactor described in Sec. II.1, containing 100 ml of distilled water and kept at *T* = 25 °C. In both pictures the upper plot is the measured CO_2_ concentration at the output of the reactor vs. time, whereas the lower plot is the pH measurement inside the reactor vs. time. (a): 200 ml total flow (80 % N_2_ + 20% CO_2_); 1 ml of tris-hydroxymethyl-aminomethane 10 mMol injected @ *t* = 80 s. (b): 200 ml total flow: N_2_ (14 min), N_2_ and 5% CO_2_ (25 min), N_2_ (25 min).

In Figure 3 two examples of measurements ([*CO*_2_]_gasOUT_ and pH /[H^+^] versus time), obtained with the two techniques, are shown. In both cases in the reactor there are 100 ml of distilled water, kept at the constant temperature *T* = 25 °C, and the constant total flow is 200 ml/min. Figure 3(a) is relative to the first technique: the input flow is composed of N_2_ and 20% CO_2_. At the time *t* = 80 s, 1 ml of buffer solution 10 mMol is injected in the reactor. In Figure 3(b), relative to the second technique, the flow composition changes: N_2_ (14 min), N_2_ and 5% CO_2_ (25 min), N_2_ (25 min). In both cases it is evident the possibility of starting a transient phase. In the lower plots of both Figure 3(a,b), the same data are reported as pH and as [*H^+^*]: from now on in the figures the information about pH will be plotted equivalently in terms of [*H^+^*] or pH.

As discussed, with both the techniques used to induce transients, the measured variables and pH are sampled using the measurement system described in Section II.1. Since the measurement system behaves as a low pass filter, and its transfer function, which also comprises the contribution of the physical system, can’t be easily predicted, this was experimentally estimated utilising a reference test. This latter consists of injecting the buffer solution standard dose, observing the measured quantities and comparing them to the theoretical ones obtained exploiting the reaction rate constants found in the literature. A first order linear system was assumed, and, to obtain the filter parameter, a model fitting was exploited.

Whatever the technique used to induce the transient, the tests were repeated with and without the CA enzyme. The experimental data were then fitted with the output of the models discussed in Sect. II.2, including the filter modelling the dynamic behaviour of the measurement system, to derive the unknown parameters of the model: i.e. the forward and backward reaction rate constants. The model implementation and the fitting procedure are described in detail in what follows.

### 2.4. Model implementation and fitting

The developed models were numerically implemented in Matlab. The differential equations were numerically solved and a non-linear least square fitting (lsqnonlin) was used to fit the experimental data and to find the unknown parameters of the model (the forward and backward reaction rate constants in Equations (4)–(6) and the filter time constant). The temperature at which the reactions occur is an input to the model, as it is kept constant by the thermostatic bath, as well as the input gas flow composition, which is derived from the flowmeter settings.

**Figure 4.**
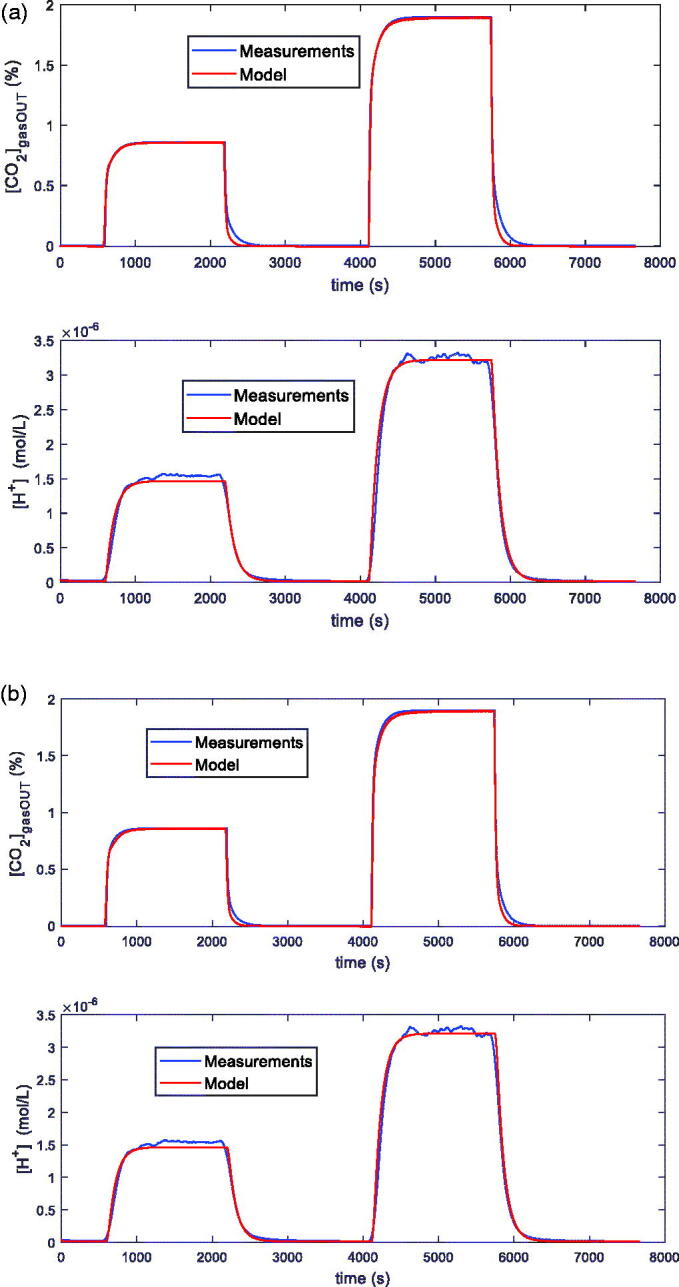
Examples of fittings obtained with the general model (a) and the simplified model (b) on data measured in dynamic conditions with the system described in Sec. II, containing 100 ml of distilled water and kept at *T* = 25 °C. In both pictures the upper plot is the measured CO_2_ concentration at the output of the reactor vs. time, whereas the lower plot is the [*H^+^*] vs. time obtained from the pH measurements. Measurement conditions: 200 ml total flow: N_2_ (10 min), N_2_ and 1% CO_2_ (25 min), N_2_ (30 min), N_2_ and 2% CO_2_ (25 min), N_2_ (30 min). Fitting maximum error lower than 3% ([*CO_2_*]) and 5% ([*H^+^*]) (a), and 3% ([*CO_2_*]) and 5% ([*H^+^*]) (b).

In particular, the fitting was obtained considering transients as proposed in this paper. In Figures 4(a,b), and in Table 1, examples of the fitting results obtained after parameter estimation for the two models, the general one and the simplified one, by using measurements in pure water with transients induced by varying the input CO_2_ concentration @*T* = 25 °C, are shown. From the curves reported in Figure 4 and from the estimated parameters shown in Table 1, it can be seen, as it was anticipated, that the simplified model is, in these conditions, accurate enough to describe the overall system transient behaviour. Therefore, from now on, the results obtained with the general model, which requires the estimation of 5 parameters instead of 4, will be shown and discussed only when they allow to obtain a significantly higher level of accuracy with respect to the simplified model.

**Table 1.** Estimated parameters of Equations (4)–(6) for the measurements shown in Figure 4(a,b). Water. General and Simplified Model.

	General model	Simplified model
*k_f0_*[1/s × 10^−2^]	4.9352	5.5612
*k_f1_*[1/s × 10^−2^]	1.7530	2.6118
*k_b_*_1_[1/s × 10^−2^]	1.4490	1.4244
*k_f2_*[1/s × 10^−3^]	0.5510	–
*k_b_*_2_[1/s]	0.096	–
*K_eq_*	–	36.7669 × 10^−4^

## 3. Results and discussion

The obtained experimental results have showed that the proposed measurement techniques can be used to characterise the activity of CA enzymes in different conditions (temperature and input CO_2_ concentration), and also that the proposed model is sufficiently accurate so as to capture the most important features of the dynamic behaviour of the system.

The results presented in this section are obtained following the measurement procedures described in the previous section at the fixed temperatures of 25 °C and 4 °C (measurements at different temperatures allow to exploit different trade offs between reaction rate, which increases with temperature, and solubility of carbon dioxide in water, which decreases with temperature). The input gas flow is always 200 ml/min, and the CO_2_ concentration is fixed and equal to 20% for transients induced by the buffer solution (first technique), and variable with values in the set (0%, 1%, 2%, 5%, 10%) for transients induced with the second technique. The experimental data are obtained both with the SspCA immobilised on the external surface of *Escherichia coli* cells^14^, and with a commercial CA from bovine erythrocytes (Sigma-Aldrich C-3934). With reference to the SspCA, the enzyme was introduced in the system using a concentration of 3.5 mg CA/g of bacterial cells.

**Figure 5.**
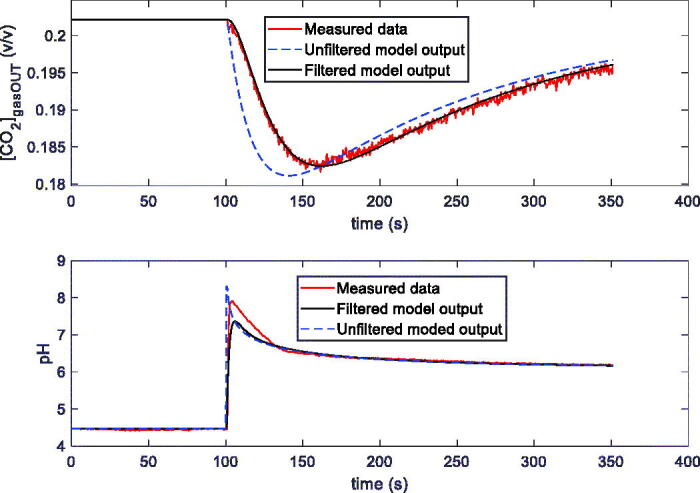
Experimental and predicted data comparison in the presence of 12 mg of membrane-bound SspCA (42 µg SspCA). Upper plot: gaseous CO_2_ concentration in the gas flow at the exhaust. Lower plot: pH vs. time. Transient induced by the buffer solution. *T* = 25 °C.

**Figure 6.**
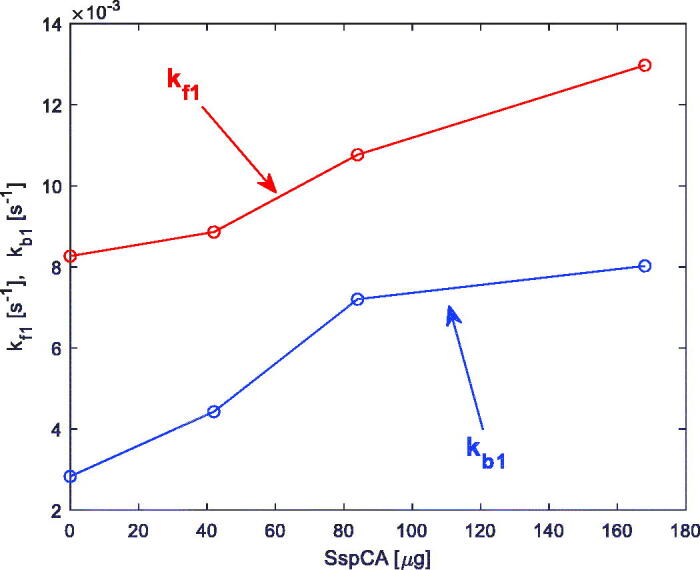
Estimated rate constants *k_f1_* and *k_b1_* (reaction (2)), obtained from the measurements described in^18^, versus SspCA quantity in the reactor.

The experimental data relative to the SspCA enzyme were already reported and discussed in^18^, and are only summarised hereafter. In particular, in Figure 5 an example of the experimental data and the prediction of the model obtained by fitting the experimental data is shown (in detail, the data are relative to the presence in the reactor of 12 mg of membrane-bound SspCA (42 µg SspCA)). The first technique was used in this case to induce a transient in the reactor, and the temperature is *T* = 25 °C. As discussed in^18^, the proposed model fits the experimental data with satisfactory accuracy. In Figure 6 a plot is shown of the estimated relevant rate constants of reaction (2), *k_f1_* and *k_b1_*, as a function of the SspCA quantity present in the reactor.

Additional experiments were performed with the CA from bovine erythrocytes inducing transients by varying the CO_2_ concentration in the input gas flow. These experiments were performed at *T* = 4 °C. The selection of this temperature is a trade off between the higher solubility of CO_2_ in water that is experienced lowering the temperature (the Bunsen coefficient almost doubles passing from *T* = 25 °C to *T* = 4 °C) and the corresponding general lowering of the reaction rate constants. Due to the different experimental conditions with respect to the experiments discussed so far, the results obtained with the general model will be considered. In any case, the worsening of the results if the simplified model is used (max 3%) does not invalidate the drawn conclusions.

**Figure 7.**
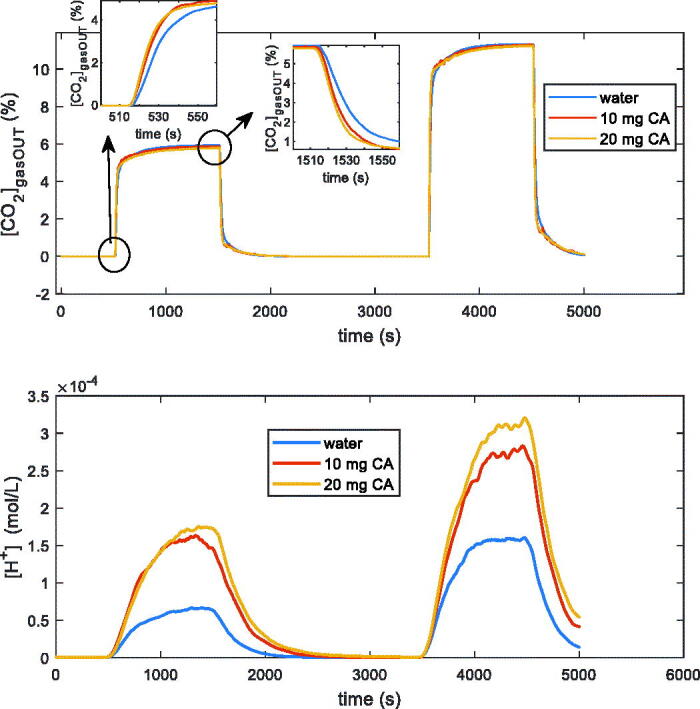
Tests in dynamic conditions with the system described in Sec. II, containing 100 ml of distilled water and kept at *T* = 4 °C. Measurement conditions: 200 ml total flow: N_2_ (8.5 min), N_2_ and 5% CO_2_ (16 min), N_2_ (32 min), N_2_ and 10% CO_2_ (16 min), N_2_ (8.5 min). The three curves are relative to water without CA and with 10 mg and 20 mg of CA from bovine erythrocytes. The upper plot shows the measured CO_2_ concentration at the output of the reactor vs. time, whereas the lower plot is the [*H^+^*] vs. time obtained from the pH measurements.

**Figure 8.**
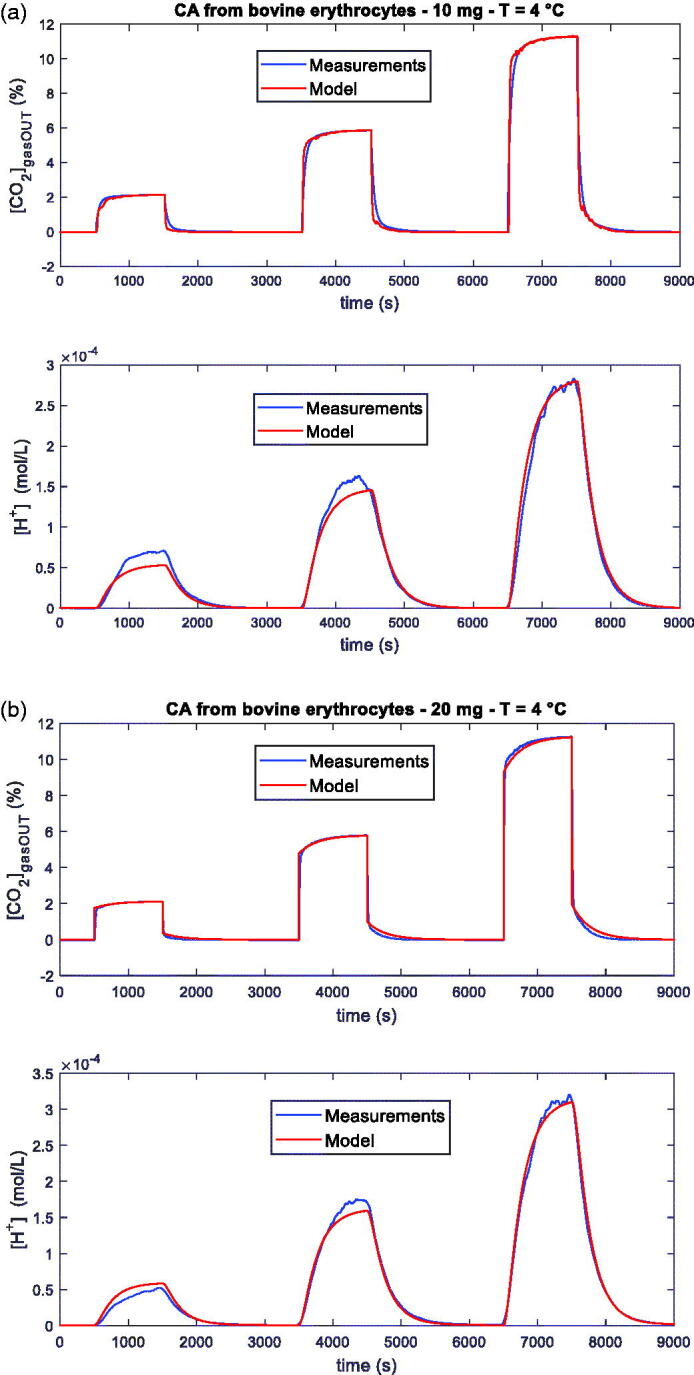
Measurements and relative fittings obtained with the general model. Measurement conditions: 200 ml total flow: N_2_ (8.5 min), N_2_ and 2% CO_2_ (16 min), N_2_ (32 min), N_2_ and 5% CO_2_ (16 min), N_2_ (32 min), N_2_ and 10% CO_2_ (16 min), N_2_ (25 min). Measurements obtained with the system described in Sec. II, containing 100 ml of distilled water, kept at *T* = 4 °C, with 10 mg (a) and 20 mg (b) of CA from bovine erythrocytes. In both pictures the upper plot is the measured CO_2_ concentration at the output of the reactor vs. time, whereas the lower plot is the [*H^+^*] vs. time obtained from the pH measurements. Fitting maximum error lower than 4% for [*CO_2_*] and 18% for [*H^+^*] (a), and 4% for [*CO_2_*] and 12% for [*H^+^*] (b).

The results obtained confirm what already discussed in^18^, and in particular that with this measurement system, together with the developed model, it is possible to characterise the catalytic activity of CA enzymes, quantifying the increase of the rate of hydration of CO_2_ also for low CA concentrations. In detail, also with the new technique to induce transients proposed in this paper, the fitting of the model (4)–(6) allows to clearly discriminate between presence and absence of CA, and among different amounts of CA (Figure 7 and Table 2). In Figure 8(a,b) the satisfactory behaviour of the model, also at *T* = 4 °C, can be appreciated.

**Table 2.** Estimated rate constants of reaction (2) for the measurements shown in Figures 7 and 8. General Model.

	*k_f1_* [1/s × 10^−3^]	*k_b1_* [1/s × 10^−3^]
Reference (no CA)	2.1789	6.8600
CA 10 mg	4.5555	10.1220
CA 20 mg	12.5110	126.6320

## 4. Conclusions

In this paper we have used chemical kinetics modelling together with dynamical system theory for interpreting measurement results obtained with a two-phase bioreactor, with the aim of characterising the activity of CA enzymes in the catalysis of the reversible reaction of the carbon dioxide (CO_2_) hydration to bicarbonate (HCO_3_^−^) and protons (H^+^). The characterisation is performed by inducing a transient (either by injecting a buffer solution or by changing the input CO_2_ gas concentration) in the bioreactor, which is the heart of the measurement system, and then monitoring and quantifying the consequent evolution of the CO_2_ hydration reaction.

In particular, the presented system, tested on commercial CA enzymes from bovine erythrocytes, allowed to obtain results proving that the proposed measurement techniques can be used to assess the efficiency of laboratory CAs like the membrane-bound SspCA, also at low concentrations. The modelling of the system under test allows for finding a robust way for deriving kinetics parameters, far less sensitive to measurement noise, in contrast to typical data processing used in the literature based on the numerical evaluation of measurement signal derivative.
